# Cobind: quantitative analysis of the genomic overlaps

**DOI:** 10.1093/bioadv/vbad104

**Published:** 2023-08-07

**Authors:** Tao Ma, Lingyun Guo, Huihuang Yan, Liguo Wang

**Affiliations:** Division of Computational Biology, Mayo Clinic College of Medicine and Science, Rochester, MN 55905, United States; Department of Computer Science and Engineering, University of Minnesota Twin Cities, Minneapolis, MN 55455, United States; Division of Computational Biology, Mayo Clinic College of Medicine and Science, Rochester, MN 55905, United States; Division of Computational Biology, Mayo Clinic College of Medicine and Science, Rochester, MN 55905, United States; Bioinformatics and Computational Biology Graduate Program, University of Minnesota Rochester, Rochester, MN 55904, United States

## Abstract

**Motivation:**

Analyzing the overlap between two sets of genomic intervals is a frequent task in the field of bioinformatics. Typically, this is accomplished by counting the number (or proportion) of overlapped regions, which applies an arbitrary threshold to determine if two genomic intervals are overlapped. By making binary calls but disregarding the magnitude of the overlap, such an approach often leads to biased, non-reproducible, and incomparable results.

**Results:**

We developed the cobind package, which incorporates six statistical measures: the Jaccard coefficient, Sørensen–Dice coefficient, Szymkiewicz–Simpson coefficient, collocation coefficient, pointwise mutual information (PMI), and normalized PMI. These measures allow for a quantitative assessment of the collocation strength between two sets of genomic intervals. To demonstrate the effectiveness of these methods, we applied them to analyze CTCF’s binding sites identified from ChIP-seq, cancer-specific open-chromatin regions (OCRs) identified from ATAC-seq of 17 cancer types, and oligodendrocytes-specific OCRs identified from scATAC-seq. Our results indicated that these new approaches effectively re-discover CTCF’s cofactors, as well as cancer-specific and oligodendrocytes-specific master regulators implicated in disease and cell type development.

**Availability and implementation:**

The cobind package is implemented in Python and freely available at https://cobind.readthedocs.io/en/latest/.

## 1 Introduction

Collocated genomic intervals indicate biological or functional connections. For example, a previous study found that over 50% of ERα-binding sites occur on FoxA1-occupied sites, and a further mechanistic study confirmed the direct collaboration between ERα and FoxA1 to control the transcription of estrogen-responsive genes (Lupien *et al.* 2008). Therefore, investigating the collocation of two sets of genomic intervals is vital in identifying common biological pathways, revealing the underlying molecular mechanisms, and validating results obtained from different experiments.

Despite its significance, the methodology for examining overlapping intervals, referred to as collocation analysis hereafter, has yet to receive extensive research attention. Currently, most researchers use an arbitrary threshold to determine if two intervals are overlapped. Based on the total number of overlaps between two interval sets, different statistical tests were employed to determine their statistical significance, such as Fisher’s exact test and odds ratio (Sheffield and Bock 2016, Layer *et al.* 2018) or permutation test (Haiminen *et al.* 2008, Ferre *et al.* 2020). This threshold-and-count approach inappropriately equates collocation analysis with co-occurrence analysis. Co-occurrence analysis is a statistical technique for measuring the joint appearance frequency of two zero-dimensional objects (e.g. gene symbols) in a dataset. The result of co-occurrence analysis can be visualized using a Venn diagram, formulated as a 2 × 2 contingency table, and the significance of co-occurrence can be evaluated using Fisher’s exact test or *χ*^2^ test. In contrast, collocation analysis deals with one-dimensional genomic intervals that have different sizes, and the magnitude of overlapping between two genomic intervals is a continuous variable. As a result, applying the co-occurrence analysis approach to genomic intervals can produce biased, non-reproducible, and incomparable results because it depends on a threshold and discards valuable information. To address these limitations, more rigorous, threshold-free approaches are needed to measure the strength of genomic collocations. To this end, we have developed the cobind package with six threshold-free metrics to objectively quantify the collocation of genomic intervals.

## 2 Methods

### 2.1 Data collection

Curated binding sites of 1208 transcription factors (TFs) were downloaded from the ReMap 2022 database (https://remap.univ-amu.fr/) (Hammal *et al.* 2022). ATAC-seq peaks of 17 TCGA common cancer types, including Bladder Urothelial Carcinoma (BLCA), Breast invasive carcinoma (BRCA), Cervical squamous cell carcinoma and endocervical adenocarcinoma (CESC), Colon adenocarcinoma (COAD), Esophageal carcinoma (ESCA), glioblastoma multiforme (GBM), Head and Neck squamous cell carcinoma (HNSC), Kidney renal clear cell carcinoma (KIRC), low-grade glioma (LGG), Liver hepatocellular carcinoma (LIHC), Lung adenocarcinoma (LUAD), Lung squamous cell carcinoma (LUSC), Prostate adenocarcinoma (PRAD), Skin Cutaneous Melanoma (SKCM), Stomach adenocarcinoma (STAD), Thyroid carcinoma (THCA), and Uterine Corpus Endometrial Carcinoma (UCEC), were downloaded from Genomics Data Commons (https://gdc.cancer.gov/about-data/publications/ATACseq-AWG) (Corces *et al.* 2018). Cancer type-specific open-chromatin regions (OCRs) were identified by comparing ATAC-seq peaks of one cancer type with the remaining cancer types using BEDTools (Quinlan and Hall 2010). Single nucleus RNA-seq (snRNA-seq) data from adult humans’ motor cortex (M1) were generated by the Allen Institute for Brain Science (https://portal.brain-map.org/) using the 10× Chromium Single Cell 3' Reagent Kit v3. Gene expression of count per million values was quantified using the default 10× Cell Ranger v3 pipeline. The University of California, Santa Cruz (UCSC) genome browser track hub to visualize these data is available at https://human-m1-rna-hub.s3-us-west-2.amazonaws.com/HumanM1RNAHub.html. Oligodendrocyte-specific single-cell assay for transposase-accessible chromatin with sequencing (scATAC-seq) peaks were downloaded from this study (Corces *et al.* 2020). Only distal peaks (*n* = 28 109) were used for collocation analysis to identify the master regulators since peaks identified from scATAC-seq are highly enriched in distal/intron regions but depleted in promoter regions.

### 2.2 The six statistical metrics

#### 2.2.1 The collocation coefficient

*A* and *B* represent two sets of genomic intervals. |*A*| represents the cardinality of *A* (i.e. all the non-redundant bases covered by *A*). |*B*| represents the cardinality of *B*. |*A* ∩ *B*| represents all the non-redundant bases covered by both *A* and *B*. The collocation coefficient (*C*) is calculated as the size of the intersection divided by the geometric mean of the two sets. *C* is defined as 0 when either of |*A*| or |*B*| is 0.


$$C\left(A,B\right)=\frac{\left|A\cap B\right|}{\sqrt{\left|A\right|\times \left|B\right|}},\;0\;\leq C\left(A,B\right)\leq 1.$$

#### 2.2.2 The Jaccard coefficient

The Jaccard coefficient is (*J*) calculated as the size of the intersection divided by the union of the two sets. *J* is defined as 0 when either of |*A*| or |*B*| is 0.


$$J\left(A,B\right)=\;\frac{\left|A\cap B\right|}{\left|A\cup B\right|}=\frac{\left|A\cap B\right|}{\left|A\right|+\left|B\right|-\left|A\cap B\right|},\;0\;\leq J\left(A,B\right)\leq 1.$$

#### 2.2.3 The Sørensen–Dice coefficient

The Sørensen–Dice (SD) coefficient is calculated as the size of the intersection divided by the arithmetic mean of the two sets. SD is defined as 0 when either of |*A*| or |*B*| is 0.


$$\mathrm{SD}\left(A,B\right)=\;\frac{\left|A\cap B\right|}{\frac{\left|A\right|+\left|B\right|}{2}},\;0\;\leq \mathrm{SD}\left(A,B\right)\leq 1.$$

The Jaccard coefficient (*J*) can be converted from SD with *J* = SD/(2−SD) and *vice versa* with SD* *=* *2*J*/(1+*J*).

#### 2.2.4 The Szymkiewicz–Simpson

The Szymkiewicz–Simpson (SS) coefficient is defined as the size of the intersection divided by the smaller size of the two sets.


$$\mathrm{SS}\left(A,B\right)=\;\frac{\left|A\cap B\right|}{\min\left(\left|A|,|B\right|\right)},\;0\;\leq \mathrm{SS}\left(A,B\right)\leq 1.$$

#### 2.2.5 Pointwise mutual information

*G* represents the size of the genome (*A* ⊂ *G* and *B* ⊂ *G*). Pointwise mutual information (PMI) is calculated as


$$\mathrm{PMI}\left(A\cap B\right)\equiv \log\left(\frac{p\left(A\cap B\right)}{p\left(A\right)\times p\left(B\right)}\right)$$

$$-\infty \leq \mathrm{PMI}(A\cap B)\leq \min\left(-\log(p(A\right)),\;-\log(p(B))),$$

where *p*(*A*), *p*(*B*), and *p*(*A∩B*) are defined as


$$p\left(A\right)=\frac{\left|A\right|}{\left|G\right|},\;p\left(B\right)=\frac{\left|B\right|}{\left|G\right|},\;p\left(A\;\cap \;B\right)=\;\frac{\left|A\cap B\right|}{\left|G\right|}.$$

#### 2.2.6 Normalized PMI

When dividing PMI by −log(*p*(*A ∩ B*)), the lower bound and upper bound are normalized to −1 and 1, respectively. Normalized PMI (NPMI) is calculated as


$$\mathrm{NPMI}\left(A\cap B\right)=\frac{\mathrm{PMI}\left(A\cap B\right)}{-\log\left(p\left(A\cap \;B\right)\right)}=\frac{\log\left(\frac{p\left(A\;\cap \;B\right)}{p\left(A\right)\times p\left(B\right)}\right)}{-\log\left(p\left(A\cap B\right)\right)}=\frac{\log\left(p\left(A\right)\times p\left(B\right)\right)}{\log\left(p\left(A\cap B\right)\right)}-1$$

$$-1\leq \mathrm{NPMI}\left(A\cap B\right)\leq 1.$$

### 2.3 Estimate confidence interval with bootstrapping

To estimate the variability and uncertainty associated with the six calculated statistical measures, we used the non-parametric bootstrapping method to estimate the 95% confidence interval. By default, 75% of input genomic intervals will be resampled and this resampling process will be repeated 20 times.

### 2.4 Conventional threshold-and-count approach to measure genomic collocations between CTCF and other TFs

Pre-analyzed, IDR (Irreproducible Discovery Rate) filtered CTCF (CCCTC-binding factor) ChIP-seq peaks (GRCh38) were downloaded from the ENCODE project (https://www.encodeproject.org/files/ENCFF660GHM/). ChIP-seq peaks from 1208 TFs were downloaded from the ReMap database (https://remap.univ-amu.fr/). The threshold-and-count approach consists of two steps: first, an arbitrary threshold (minimum number of nucleotides overlapped) was used to determine the overlapping of two peaks, and then each CTCF peak was labeled by “0” or “1”, representing non-overlap or overlap with peaks of the TF compared, respectively; second, the percentage of overlapped CTCF peaks was calculated as the measure of collocation strength. This approach was evaluated using six different thresholds: 1-nucleotide (the most relaxed), 10%, 30%, 50%, 80%, and 100% (the most stringent). Based on the percentage of overlapped CTCF peaks, the 1207 TFs (CTCF itself was excluded) were ranked in descending order under each criterion.

### 2.5 Calculate the combined *Z*-score

We used the Gene Set Variation Analysis package (Hänzelmann *et al.* 2013) to calculate the combined *Z*-scores. Specifically, scores of the six metrics were converted into *Z*-scores by *Z*_i_ = (*x* − *μ*)/*σ*, where *μ* and *σ* are the average and standard deviation of the score, and *i* ∈ {*C*, *J*, SD, SS, PMI, NPMI}. The combined *Z*-score is defined as:


$$Z=\frac{\sum Z_{i}}{\sqrt{6}}.$$

TFs with *Z*-scores that are more than 3 standard deviations away from the mean are considered outliers (i.e. potential master regulators).

## 3 Results

### 3.1 Overview of collocation metrics

The collocation coefficient (*C*), the Jaccard coefficient (*J*), the Sørensen–Dice coefficient (SD), and the Szymkiewicz–Simpson coefficient (SS) are intersection-based metrics and conceptually similar: they normalize the size of the intersection between *A* and *B* by the “geometric mean,” “union,” “arithmetic mean,” and “min” of *A* and *B* (*A* and *B* represent two sets of genomic intervals), respectively. As can be seen from Section 2 and Table 1, all the intersection-based coefficients are bounded by 0 (no collocation) and 1 (complete collocation), which facilitate cross-sample comparison and integration. In theory, *J* and SD scores are prone to be biased by the larger interval set due to their use of “union” and “arithmetic mean” as denominators, respectively. Conversely, SS is susceptible to the smaller interval set as it uses “min” as the denominator. However, the novel metric *C* developed in this study utilizes the geometric mean that is less sensitive to highly skewed data, thus could alleviate the observed bias in *J*, SD, and SS (Supplementary Fig. S1; Section 2).

**Table 1.** The six collocation measurements and their lower and upper bounds.

Metrics	Nominator	Denominator	Lower bound (when *A* ∩ *B* = 0)	Upper bound (when *A* = *B*)
*C*	|A∩B|	$\sqrt{\left|A|\times |B\right|}$	0	1
*J*	|A∩B|	|A∪B|	0	1
SD	|A∩B|	(|A| + |B|)/2	0	1
SS	|A∩B|	$\mathrm{Min}(\left|A\right|,\;\left|B\right|)$	0	1 (when *A* = *B*, *A* ∈ *B*, *B* ∈ *A*)
PMI	See Section 2		−∞	min(−log(*p*(*A*)), −log(*p*(*B*)))
NPMI	See Section 2		−1	1

*Notes*: *A* and *B* represent two sets of genomic intervals. *C*, the collocation coefficient; *J*, the Jaccard coefficient; *SD*, the Sørensen–Dice coefficient; SS, the Szymkiewicz–Simpson coefficient.

In addition to these four intersection-based metrics, we also introduced two information-theory-based association measures: PMI and normalized PMI (NPMI). PMI measures how much the observed overlap differs from what we expected. PMI =* *0 indicates that *A* and *B* are independent, whereas PMI > 0 and PMI < 0 indicate that the overlap between *A* and *B* is higher and lower than expected if *A* and *B* were independent, respectively. Although PMI is a standard measure to quantify overlapping or co-occurrence, the lack of a fixed upper bound compromises its use for cross-sample comparison. Instead, NPMI gives a fixed upper bound and lower bound with “−1” and “1,” indicating no collocation and complete collocation, respectively (Table 1).

### 3.2 Identify TFs co-localized with CTCF bindings

**Figure 1.**
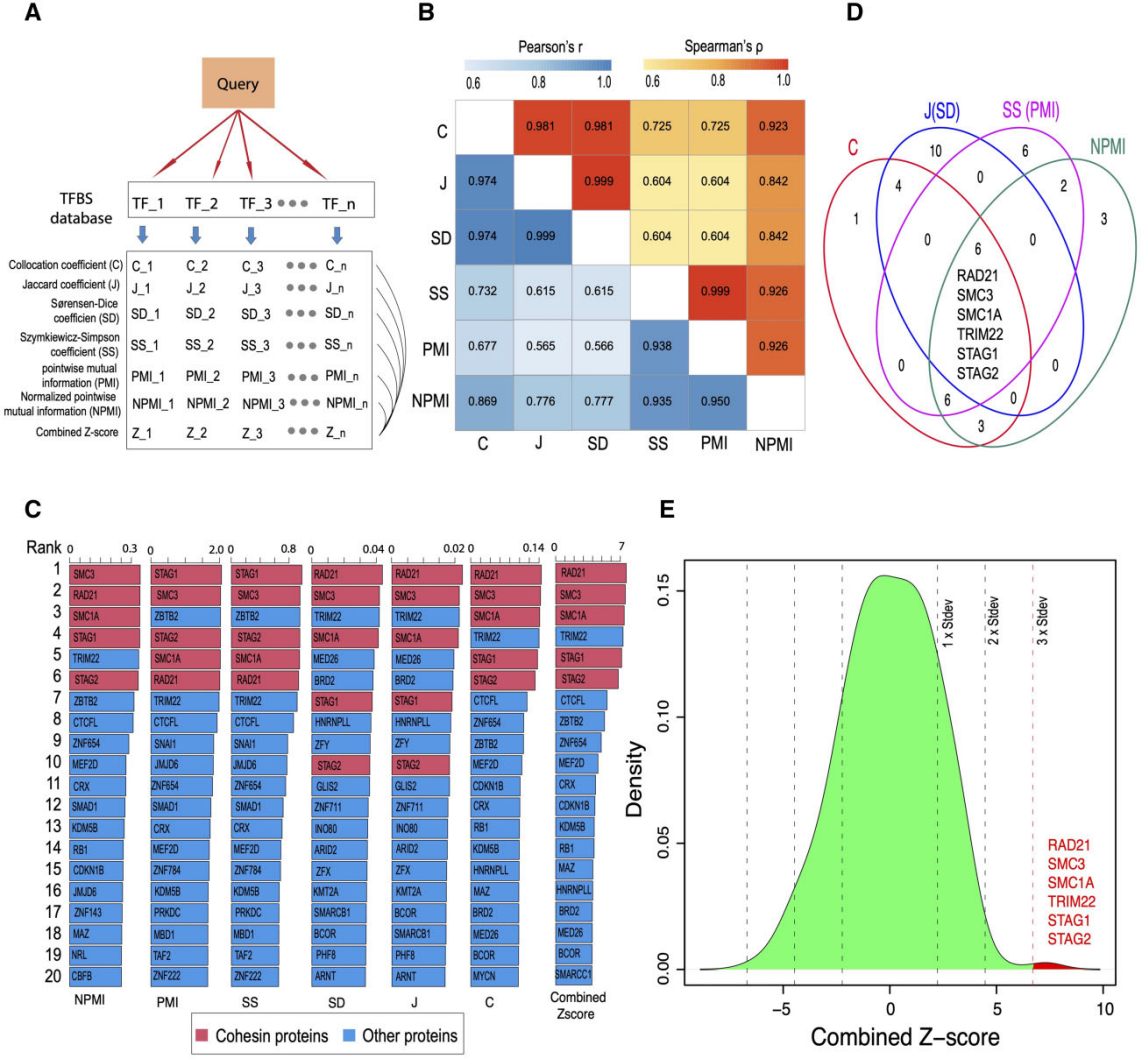
Evaluation of the collocation metrics implemented in cobind using CTCF binding sites. (A) The process of calculating the six collocation measurements (and the combined *Z*-score) of a query genomic interval with the database of TFBS. (B) Heatmap shows Pearson’s and Spearman’s pairwise correlations between six collocation metrics, including *C*, *J*, SD, NPMI, PMI, and SS. The collocation coefficients were calculated between CTCF binding sites and the binding sites of 1207 TFs. (C) Barplots show the top 20 TFs whose binding sites overlap with CTCF binding sites. Outliers (defined as TFs whose *Z*-score is at least three standard deviations from the mean) were filled in red, while other proteins were filled in blue. (D) Venn diagram shows the overlaps of the top 10 master regulators reported by *C*, *J*(SD), SS(PMI), and NPMI. *J* and SD, SS, and PMI were combined since the top 10 master regulators were the same. (E) The density plot shows the five cohesin proteins and TRIM22 were identified as outliers. *C*, collocation coefficient; *J*, the Jaccard coefficient; SD, the Sørensen–Dice coefficient; SS, the Szymkiewicz–Simpson coefficient; PMI, pointwise mutual information; NPMI, normalized pointwise mutual information.

CTCF is a TF that plays a critical role in chromatin organization. The interdependence between CTCF and the cohesin proteins (including RAD21, SMC1A, SMC3, STAG1, and STAG2) to regulate the 3D chromatin architecture is well-known and has been independently validated by numerous studies (Hansen *et al.* 2017, Cattoglio *et al.* 2019, Li *et al.* 2020, Pugacheva *et al.* 2020, Gabriele *et al.* 2022, Mach *et al.* 2022). Their collaborative relationship leads to collocated bindings. This allows us to evaluate the performance of the six collocation measurements implemented in the cobind package. We first calculated the scores of these metrics between CTCF’s binding sites (CBF) and those binding sites of other 1207 TFs curated in the ReMap database (https://remap.univ-amu.fr/). We also calculated *Z*-scores combining all six metrics as an overall measure (Fig. 1A and Supplementary Table S1; Section 2).

The results indicated that the six metrics exhibited high pairwise concordance with Spearman’s correlation coefficient (*ρ*) ranging from 0.60 to 0.99 (mean = 0.83, median = 0.88) and Pearson’s *r* ranging from 0.57 to 0.99 (mean = 0.80, median = 0.78) (Fig. 1B). All six metrics successfully ranked the five cohesin proteins (RAD21, SMC1A, SMC3, STAG1, STAG2) and the TRIM22 protein in the top 10 (Fig. 1C and D). Considering data points with more than 3 standard deviations from the mean as outliers, these six proteins were the only outliers detected (Fig. 1E). This suggests that their collocations with CTCF are significantly higher than those of other proteins. Although the exact mechanism is unclear, multiple studies have identified TRIM22 as a critical regulator of chromatin structure. TRIM22 bindings are highly enriched at chromatin contact domain boundaries (Weintraub *et al.* 2017, Chen *et al.* 2018). TRIM22 is reported as one of the most informative proteins for predicting human chromosome structures (Di Pierro *et al.* 2017). TRIM22 has also been identified as one of the loop-mediating proteins by a deep learning algorithm (RefHiC) using HiC data (Zhang and Blanchette 2022). In summary, these algorithms could successfully nominate CTCF’s cofactors from other DNA-binding proteins and highlight the potential of collocation analysis in revealing meaningful biological associations.

As a comparison, the conventional threshold-and-count approach missed the majority of cohesin proteins from the top 10 rankings, regardless of the thresholds applied (Supplementary Fig. S2). We found that only one cohesin protein (RAD21) was identified with relatively relaxed thresholds (i.e. from 1 nt overlap to 30% of CTCF peak overlap). When using the most stringent thresholds (>50% of CTCF peak overlap), two to three cohesin proteins (RAD21, SMC1A, and SMC3) were detected (Supplementary Fig. S2). These results indicate that the threshold-and-count approach is influenced by the number of peaks and lacks the sensitivity to identify CTCF’s cofactors. For example, this method consistently ranked BRD4 and ESR1 at the top of the list. These two TFs are likely false positives; they are ranked the highest simply because BRD4 (1105211 peaks) and ESR1 (866869 peaks) have the largest and the second largest number of peaks, respectively.

### 3.3 Identify the master regulators co-localized with cancer-specific OCRs

OCRs refer to exposed DNA accessible to TFs and other regulatory proteins. Collocation analysis of TF binding sites (TFBS) with cancer-specific OCRs offers the opportunity to identify master regulators implicated in modulating cancer-specific transcription programs. To demonstrate the utility of our approach, we applied the algorithms implemented in the cobind package to analyze the collocation between the TFBS of 1208 TFs (Supplementary Table S1) and OCRs specific to 17 common cancers (Supplementary Table S2). The resulting collocation *Z*-scores are listed in Supplementary Table S3.

**Figure 2.**
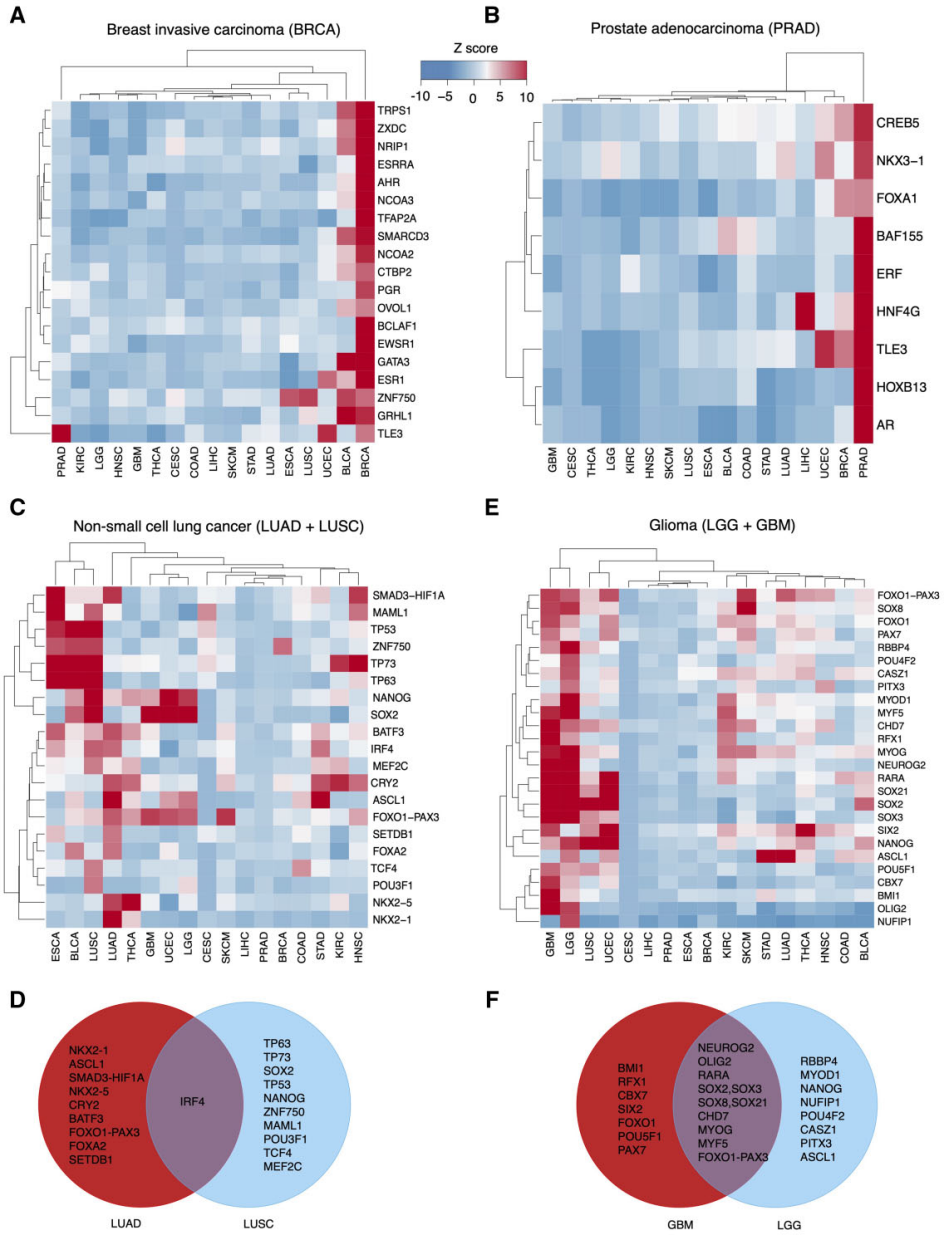
Evaluation of the collocation metrics implemented in cobind using cancer-specific OCRs identified from ATAC-seq data. (A) Candidate master regulators identified from breast cancer-specific OCRs. (B) Candidate master regulators identified from prostate cancer-specific OCRs. (C) Candidate master regulators identified from LUAD- and LUSC-specific OCRs. (D) Venn diagram shows the overlap of master regulators identified from LUAD and LUSC. (E) Candidate master regulators identified from LGG and GBM. (F) Venn diagram shows the overlap of master regulators identified in LGG and GBM.

Our collocation analyses revealed many well-known, cancer-specific master regulators (Supplementary Fig. S3 and Supplementary Tables S3 and S4). For example, GATA3, ESR1, and TRPS1 were identified as the three leading master regulators in breast cancer (Fig. 2A). The implications of these TFs with breast cancer are reported by numerous studies (Witwicki *et al.* 2018, Dustin *et al.* 2019, Brett *et al.* 2021, Herzog and Fuqua 2022, Parkinson *et al.* 2022, Yoon *et al.* 2022). AR, HOXB13, and TLE3 were identified as the top three master regulators in prostate cancer (Fig. 2B), which is also in line with previous findings (Heinlein and Chang 2004, Palit *et al.* 2019, Jamroze *et al.* 2021, Darst *et al.* 2022, Lu *et al.* 2022). SOX10, a key nuclear TF involved in differentiating neural crest progenitor cells into melanocytes, was recognized as the top master regulator in Skin Cutaneous Melanoma (SKCM) (Rosenbaum *et al.* 2021, Capparelli *et al.* 2022). CDX2, an intestine-specific TF that regulates intestinal development and oncogenesis, was found as the top master regulator in colon adenocarcinoma (COAD) (Crissey *et al.* 2011, Pilati *et al.* 2017, Yu *et al.* 2019). Lung adenocarcinoma (LUAD) and lung squamous cell carcinoma (LUSC) are the most common subtypes of lung cancer. Although both LUAD and LUSC are classified as non-small cell lung cancer, they have drastically different biology, etiology, molecular characteristics, and clinical behavior. Our collocation analysis revealed that NKX2-1 (Winslow *et al.* 2011, Mollaoglu *et al.* 2018, Phelps *et al.* 2018, Ingram *et al.* 2022) and ASCL1 (Kosari *et al.* 2014, Baine *et al.* 2020) are the top regulators in LUAD, while TP63, TP73, SOX2, and TP53 are the top regulators in LUSC. In fact, we only observed one common TF, IRF4, which regulates transcription programs in immune cells, out of the 10 and 11 master regulators in LUAD and LUSC, respectively (Fig. 2C and D). LGG and GBM are two common brain tumor types. LGG is slow growing, has a better prognosis and response to treatment, and tends to recur less frequently. GBM, on the other hand, is more aggressive, has a poorer prognosis with a shorter overall survival rate and a higher recurrence rate. Our collocation analyses revealed 19 master regulators in LGG and 18 master regulators in GBM, of which 11 (NEUROG2, OLIG2, RARA, SOX2, SOX3, SOX8, SOX21, CHD7, MYOG, MYF5, FOXO1-PAX3) are shared (Fig. 2E and F). Of note, BMI1 was identified as one of the top master regulators in GBM but not in LGG, and numerous studies have revealed that BMI1 plays a significant role in promoting cell proliferation, inhibiting cell death, sustaining stem cell renewal, and activating signaling pathways, all of which contribute to the development, progression, and aggressiveness of GBMs (Venugopal *et al.* 2012, Vora *et al.* 2019, Flamier *et al.* 2020, Freire-Benéitez *et al.* 2021). In summary, our analyses suggested that collocation analyses of cancer-specific OCRs with TFBS can provide valuable information for understanding the molecular mechanisms that drive cancer development and identify biomarkers for cancer diagnosis, prognosis, and treatment.

### 3.4 Identify the master regulators co-localized with oligodendrocyte-specific open chromatin

In a study profiling the chromatin accessibility of more than 70 000 human adult brain cells, Corces *et al.* (2020) discovered six major cell types, including oligodendrocytes, excitatory neurons, inhibitory neurons, microglia, astrocytes, and oligodendrocyte progenitor cells, of which oligodendrocytes is the largest cell population. To identify putative master regulators responsible for establishing and maintaining cell type-specific regulatory programs, the authors performed DNA motif enrichment analyses on scATAC-seq peak regions specific to oligodendrocytes. They found that SOX9, SOX10, and FOXN2 DNA motifs were significantly enriched in the oligodendrocytes OCRs (Corces *et al.* 2020).

**Figure 3.**
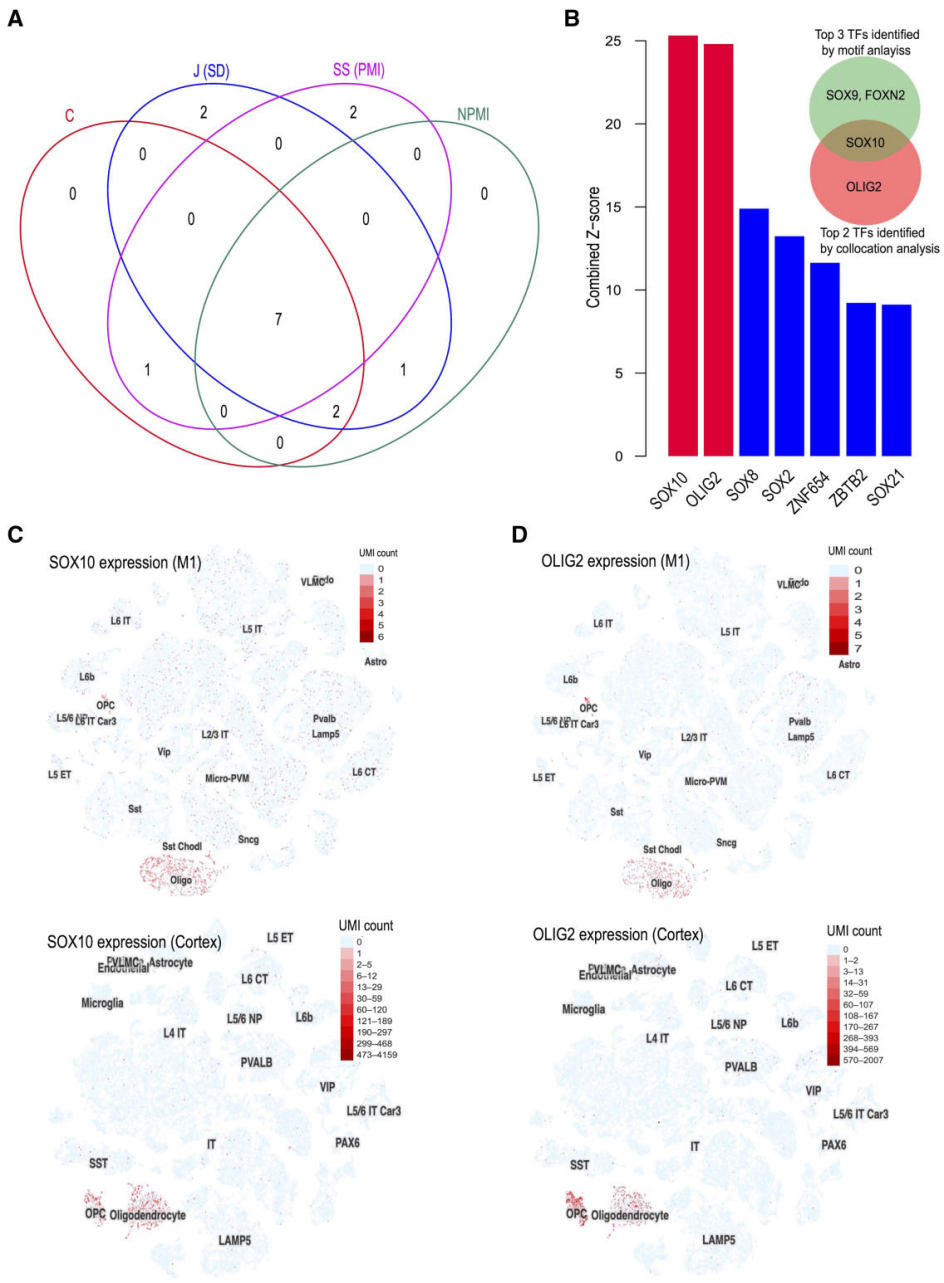
Evaluation of the collocation metrics implemented in cobind using oligodendrocytes-specific OCRs identified from scATAC-seq data. (A) Venn diagram shows the overlaps of the top 10 master regulators reported by *C*, *J*(SD), SS(PMI), and NPMI. *J* and SD, SS, and PMI were combined since the top 10 master regulators were the same. (B) Barplot shows the *Z*-scores of the seven master regulators reported by all six metrics. Venn diagram shows the overlap between the top three TFs identified by motif analysis (green circle) and the top two TFs identified by collocation analysis (red circle). (C) t-distributed stochastic neighbor embedding (tSNE)-based cell map shows that *SOX10* is specifically expressed in oligodendrocytes. Upper: *SOX10* expression in human primary motor cortex region (M1). Lower: *SOX10* expression in human cortex brain region. (D) tSNE-based cell map shows that *OLIG2* is specifically expressed in oligodendrocytes. Upper: *OLIG2* expression in the human primary motor cortex (M1) region. Lower: *OLIG2* expression in the human cortex brain region. UMI, unique molecular identifiers.

Aiming to identify the master regulators in oligodendrocytes, we used the cobind package to conduct the collocation analysis between oligodendrocyte-specific OCRs and the binding sites of 1208 TFs (Supplementary Table S5). Our methods revealed seven TFs (SOX10, OLIG2, SOX8, SOX2, ZNF654, ZBTB2, and SOX21) that were significantly collocated with oligodendrocyte-specific *cis*-regulatory regions (Fig. 3A and Supplementary Fig. S4), among which SOX10 and oligodendrocyte TF 2 (OLIG2) had the highest *Z*-scores (Fig. 3B), suggesting that they are master regulators of oligodendrocytes. To independently validate these results, we examined the snRNA-seq expression data generated from the human cortex and primary motor cortex region (M1) by the Allen Brain Map project (https://portal.brain-map.org/). Indeed, we observed that SOX10 and OLIG2 were expressed specifically in oligodendrocytes (Fig. 3C and D).

Among the three TFs (SOX10, SOX9, and FOXN2) reported by Corces *et al.* (2020) using the motif enrichment method, two (SOX9 and FOXN2) were not detected by our collocation analysis approach; SOX9 was ranked 89th by *Z*-score, while FOXN2 was not included in the ReMap database (Supplementary Table S5). snRNA-seq data show that SOX9 and FOXN2 genes were not explicitly expressed in oligodendrocytes, suggesting these two TFs are less likely to be master regulators (Supplementary Fig. S5). In contrast, OLIG2, which was missed by the motif enrichment analysis method, is explicitly expressed in both oligodendrocytes and oligodendrocyte progenitor cells (Fig. 3B and D) and has proven to play an imperative role in promoting oligodendrocyte differentiation (Takebayashi *et al.* 2000, Zhou *et al.* 2001, Yu *et al.* 2013). These results highlighted that collocation analysis is a more robust approach to revealing key TFs that might be missed by motif analysis.

## 4 Summary and discussion

The metrics implemented in the cobind package (except the collocation coefficient) have been widely utilized in the Natural Language Processing field to determine if two words occur more frequently than expected by chance. However, in the genomics field, only the Jaccard coefficient has been adopted by BEDTools to measure the overlaps between two BED files. It is worth noting that there is a slight difference in the calculation of the Jaccard coefficient between cobind and BEDTools. Cobind considers every input genomic interval, regardless of overlap, while BEDTools only consider intervals that exhibit overlap.

Motif analysis, a computational approach to identify overrepresented short-nucleotide patterns, has been extensively applied to identify common regulators from a given set of genomic intervals. Although DNA motif analysis can provide valuable insights, it is prone to false positives and false negatives since most TFs only bind less than a few percent of their consensus target sites in the genome (Zaret and Carroll 2011). In our study, we demonstrated that collocation analysis successfully identified a known master regulator in oligodendrocytes, OLIG2, which was not detected by motif analysis. This result highlights the collocation analysis’s potential as a more effective alternative to motif analysis. However, collocation analysis necessitates a comprehensive list of well-annotated *cis*-regulatory sites. Fortunately, public consortia such as ENCODE, modENCODE, Roadmap, and functional genomic repositories like GEO (https://www.ncbi.nlm.nih.gov/geo/) offer rich resources encompassing a wide range of TFs from numerous tissues and cell lines.

The cobind package is the first bioinformatics tool specifically designed for quantitative collocation analysis of genomic intervals. It is efficient in terms of speed and memory usage (Supplementary Table S6). This study demonstrated the significant potential of cobind in characterizing the functionalities of noncoding genomic regions, integrating multi-omics data, and gaining new biological insights. This potential is particularly relevant in the context of functional genomics assays such as scATAC-seq, which can reveal cell type-specific regulatory elements.

## Supplementary Material

vbad104_Supplementary_Data

## Data Availability

The data underlying this article are available in the article and in its online supplementary material.
