# Supplementary material for: Cobind: quantitative analysis of the genomic overlaps
Source: Bioinform Adv. 2023 Aug 7;3(1):vbad104. doi: 10.1093/bioadv/vbad104 (PMC10438957; doi:10.1093/bioadv/vbad104)
Supplement: vbad104_Supplementary_Data [file vbad104_supplementary_data.zip › Supplementary Figures.docx]

**Supplementary Figures**

**Cobind: quantitative analysis of the genomic overlaps**

## Tao Ma^1^, Lingyun Guo^2^, HuiHuang Yan^1^ and Liguo Wang^1,3,*^

^1^ Division of Computational Biology, Mayo Clinic College of Medicine and Science, Rochester, MN 55905, USA

^2^ Department of Computer Science and Engineering, University of Minnesota Twin Cities, Minneapolis, MN 55455, USA

^3^ Bioinformatics and Computational Biology Graduate Program, University of Minnesota Rochester, Rochester, MN 55904, USA

*To whom correspondence should be addressed.

**Supplementary Figure S1: Simulated collocation scores of the four intersection-based metrics (*C*, *J*, *SD*, and *SS*) between two sets of genomic intervals *M* and *N*.** (A) Assume *M* = 1000, *N* = 1000, and |*M* ∩ N|=1000 (i.e. M and N are completely collocated). Then, we increased the size of *M* from 1000 to 10000 to evaluate how the imbalances impact the scores of *C*, *J*, *SD*, and *SS*. (B) Assume *M* = 10000, *N* = 10000, and |*M* ∩ *N|*=1000. Then we decrease the size of M from 10000 to 1000 to evaluate how the imbalances impact the scores of *C*, *J*, *SD*, and *SS*.

**Supplementary Figure S2: The top 100 transcription factors ranked from high to low based on the percentages of CTCF binding sites that overlapped with the ChIP-seq peaks of each TF.** The overlap percentages (y-axis) are calculated using the threshold-and-count approach with seven different thresholds: (A) 1-nucleotide, (B) 10%, (C) 20%, (D) 30%, (E) 50%, (F) 80%, and (G) 100%. The cohesin proteins are highlighted in red.

**Supplementary Figure S3: Z-score heatmap shows master regulators identified from cancer-specific open chromatin regions (OCRs). Rows represent 190 transcription factors whose Z-score is greater than 3 standard deviations from the mean.** Columns represent 17 common cancer types including BLCA, Bladder Urothelial Carcinoma; BRCA, Breast invasive carcinoma; CESC, Cervical squamous cell carcinoma and endocervical adenocarcinoma; COAD, Colon adenocarcinoma; ESCA, Esophageal carcinoma; GBM, Glioblastoma multiforme; HNSC, Head and Neck squamous cell carcinoma; KIRC, Kidney renal clear cell carcinoma; LGG, Brain Lower Grade Glioma; LIHC, Liver hepatocellular carcinoma; LUAD, Lung adenocarcinoma; LUSC, Lung squamous cell carcinoma; PRAD, Prostate adenocarcinoma; SKCM, Skin Cutaneous Melanoma; STAD, Stomach adenocarcinoma; THCA, Thyroid carcinoma; UCEC, Uterine Corpus Endometrial Carcinoma.

**Supplementary Figure S4: Barplot shows the top 10 master regulators identified from oligodendrocytes-specific open chromatin regions (OCRs) by six different metrics.** Transcription factors shared by all the metrics are highlighted in red. *C*, collocation coefficient; *J*, The Jaccard coefficient; *SD*, the Sørensen-Dice coefficient; *SS*, the Szymkiewicz-Simpson coefficient; *PMI*, pointwise mutual information; *NPMI*, normalized pointwise mutual information.

**Supplementary Figure S5: The tSNE (t-distributed stochastic neighbor embedding) based cell map shows *SOX9* and *FOXN2* expressions in the human primary motor cortex (M1) region (upper panel) and cortex brain region (low panel).** UMI, unique molecular identifiers.
